# Recruitment-to-inflation ratio measured with modern intensive care unit ventilators: How accurate is it?

**DOI:** 10.1186/s13054-022-03961-x

**Published:** 2022-03-28

**Authors:** Martin Cour, Charlotte Biscarrat, Neven Stevic, Florian Degivry, Laurent Argaud, Claude Guérin

**Affiliations:** 1 Service de Médecine Intensive-Réanimation, Hôpital Edouard Herriot, Hospices Civils de Lyon, 5, place d’Arsonval, 69437 Lyon Cedex 03, France; 2 Faculté de médecine Lyon-Est, Université de Lyon, Université Claude Bernard Lyon 1, 69373 Lyon, France

The recruitment-to-inflation (*R*/*I*) ratio is a recent tool that has been developed to evaluate the potential for lung recruitment in patients with acute respiratory distress syndrome (ARDS) [1]. It is calculated as the ratio between the compliance of the recruited lung following the application of a high positive end expiratory pressure (PEEP) to that of the respiratory system measured at lower PEEP. This parameter can be easily measured at bedside with any intensive care unit (ICU) ventilator [1]. Identifying ARDS patients with high potential for lung recruitment is important to help choosing ventilatory settings, particularly the PEEP level [1]. In the landmark study by Chen et al. [1], a cut-off value of *R*/*I* at 0.5, i.e. the median value of the cohort, was proposed to define patients with low (*R*/*I* ≤ 0.5) or high (*R*/*I* > 0.5) potential for lung recruitment. Since then, the *R*/*I* ratio has been used for phenotyping ARDS [2] or to assess the effects of interventions (e.g. prone positioning [3], lung recruitment maneuvers [4]) according to the potential for lung recruitment. Before using this very promising tool at a large scale in trials, it is important to verify the accuracy and the consistency of its values across the ICU ventilators. Indeed, errors in measures of both volumes and pressures are common with most modern ICU ventilators, even after careful calibration, and often excess 10% [5]. In the present bench study, we aimed to assess accuracy of modern ICU ventilators for measuring *R*/*I* ratio set at 0.0, 0.5 and 1.0 on a lung model simulator.

We used an ASL-5000 lung model (Ingmar Medical, Pittsburgh, PA) to simulate PEEP-induced recruited lung volume (Vrec) by modifying the compliance of the lung model at high PEEP, in order to obtain *R*/*I* ratios equal to 0.0, 0.5 or 1.0. At low PEEP, the compliance of the test lung was set at 40 ml/cmH_2_O (i.e., a common value in ARDS). At high PEEP, the compliance was either unchanged (40 ml/cmH_2_O) or increased to 60 or 80 ml/cmH_2_O to obtain the abovementioned *R*/*I* ratios. Thus, for a 10 cmH_2_O difference between low and high PEEP, the expected Vrec was 0, 200 and 400 ml, respectively.

Five modern ICU ventilators were assessed: Carestation (General Electric, Fairfield, CO), Servo I (Maquet, Solna, Sweden), Hamilton C5 (Hamilton, Rhäzuns, Switzerland), Infinity C500 (Dräger, Lübeck, Germany) and Evita XL (Dräger, Lübeck, Germany). Ventilators were fully checked and calibrated according to the manufacturers’ specifications. The Y-piece of the double-limb ventilator tubing was directly connected to the ASL-5000. Ventilators were set in volume control mode with tidal volume (*V*_T_) 400 ml, inspiratory flow 60 l/min, respiratory rate 20 breaths/min, and F_I_O_2_ 21%. The low and high PEEP were set at 5 and 15 cmH_2_O, respectively. *R*/*I* ratios were calculated from the data measured by the pressure transducers and flowmeters of the respirators, as previously described [1]. They included plateau pressure, PEEP total, expired *V*_T_*,* and end-expiratory lung volume change when PEEP was abruptly decreased from 15 to 5 cmH_2_O on a single breath (Table 1). Measures of parameters needed to calculate *R*/*I* ratio were performed twice with each ventilator. The differences between the measured and the theoretical *R*/*I* ratios were calculated for each ventilator.

**Table 1:** Measures of *R*/*I* ratio with 5 ventilators according to theoretical *R*/*I* ratio equal to 0.0, 0.5 and 1.0

Theoretical *R*/*I* ratio	Ventilator	PEEP 5 cmH_2_O			PEEP 15 cmH_2_O			PEEP 15-to-5	Calculated values		
		VTe (ml)	PPlat (cmH_2_O)	PEEPt (cmH_2_O)	VTe (ml)	PPlat (cmH_2_O)	PEEPt (cmH_2_O)	EELV (ml)	Vrec (ml)	*R*/*I*	Error in *R*/*I*
0.0	A	428	14	5	412	23	15	921	33	0.07	+ 0.07
		429	14	5	413	24	15	921	28	0.06	+ 0.06
	B	397	14	5	386	23	15	831	4	0.01	+ 0.01
		385	14	5	381	23	15	825	16	0.04	+ 0.04
	C	404	14.3	5.2	407	23.8	15.2	898	47	0.11	+ 0.11
		402	14.4	5.3	397	24.2	15.4	877	34	0.08	+ 0.08
	D	350	14.9	5.4	353	24.5	15.3	747	29	0.08	+ 0.08
		358	14.7	5.4	354	24.2	15.4	776	37	0.10	+ 0.10
	E	370	14	6	380	24	15	797	1	0.00	+ 0.00
		385	14	6	381	24	15	790	− 24	− 0.05	− 0.05
**0.5**	A	420	14	5	419	21	15	1172	286	0.61	+ 0.11
		429	14	5	414	21	15	1162	271	0.57	+ 0.07
	B	397	14	5	392	21	15	1060	227	0.51	+ 0.01
		385	14	5	384	21	15	1045	223	0.55	+ 0.05
	C	404	14.3	5.2	405	21.8	15.6	1144	277	0.60	+ 0.10
		402	14.4	5.3	398	21.0	15.1	1121	290	0.67	+ 0.17
	D	350	14.9	5.4	350	22.5	15.9	977	230	0.59	+ 0.09
		358	14.7	5.4	355	22.0	15.9	951	192	0.47	− 0.03
	E	370	14	6	365	22	16	950	123	0.26	− 0.24
		385	14	6	385	22	16	1010	144	0.30	− 0.20
**1.0**	A	428	14	5	416	20	16	1408	471	0.90	− 0.10
		429	14	5	415	20	16	1406	467	0.89	− 0.11
	B	397	14	5	393	20	15	1269	435	0.99	− 0.01
		385	14	5	385	20	15	1258	445	1.04	+ 0.04
	C	404	14.3	5.2	397	20.4	15.9	1365	493	1.03	+ 0.03
		402	14.4	5.3	394	20.6	15.9	1322	460	0.98	− 0.02
	D	350	14.9	5.4	353	22.1	16.4	1140	382	0.94	− 0.06
		358	14.7	5.4	351	21.0	16.5	1137	359	0.84	− 0.16
	E	370	14	6	387	21	16	1220	370	0.80	− 0.20
		385	14	6	385	20	16	1220	353	0.73	− 0.27

Values are expressed as number and reported as displayed by the ventilator (i.e. with or without a decimal expansion). The ventilators were set in volume control mode with a tidal volume of 400 ml. Two independent set of measures are reported for each of the 5 ventilators (A: Carestation, B: Servo I, C: Hamilton C5, D: Infinity C500; E: EVITA XL)

*R*/*I*, recruitment-to-inflation ratio; PEEP, positive end-expiratory pressure; PEEP 15-to-5, decrease in PEEP from 15 to 5 cmH_2_O on a single breath; VTe, expired tidal volume; PPlat, plateau pressure; PEEPt, total PEEP; EELV, end-expiratory lung volume; Vrec, recruited lung volume

As shown in Table 1, *R*/*I* ratios were overestimated by 4/5 of the ventilators for theoretical *R*/*I* of 0.0 or 0.5 and underestimated by 3/5 of the ventilators for theoretical *R*/*I* of 1.0 (Table 1). For the theoretical *R*/*I* = 0.5 (i.e. the value commonly used in the literature to discriminate recruiters and non-recruiters), the error in the measured *R*/*I* was > 0.05 (> 10%) with 4/5 ventilators and > 0.1 (> 20%) with 3/5 ventilators (Table 1). For this condition, the highest overestimation of *R*/*I* was + 0.17 (+ 34%), the highest underestimation of *R*/*I* was − 0.24 (− 48%) and the highest error in Vrec was + 90 ml (+ 45%). The highest difference in *R*/*I* between 2 ventilators was 0.4.

The present study highlighted that clinically relevant underestimations or overestimations of the *R*/*I* ratio and/or of the Vrec are common when measured with modern ICU ventilators. Several clinical studies used a single cut-off value of *R*/*I* to discriminate groups of patients with low or high potential for lung recruitment [1–4]. However, our results showing large errors in the measurements of the true values of *R*/*I*, despite highly standardized bench conditions, suggest that using a single cut-off *R*/*I* value to individualize treatments of a given patient may be inappropriate and could even lead to opposite therapeutic strategies.

Clinicians should be aware of a range of values around a given cut-off *R*/*I* value (i.e. a grey zone) for which no conclusion may be drawn concerning potential for lung recruitment, especially if they use different models of ventilators in the same ICU. These insights need to be taken into account for interpreting and/or designing future studies on *R*/*I* ratios.

## Data Availability

The data that support the findings of this study are available from the corresponding author. The research team will provide an email address for communication of the data.

## Abbreviations

ARDS: Acute respiratory distress syndrome
ICU: Intensive care unit
PEEP: Positive end expiratory pressure
*R*/*I*: Recruitment-to-inflation ratio
Vrec: Recruited lung volume
